# Diagnosis of partial complex regional pain syndrome type 1 of the hand: retrospective study of 16 cases and literature review

**DOI:** 10.1186/1471-2377-13-28

**Published:** 2013-03-18

**Authors:** Michel Konzelmann, Olivier Deriaz, François Luthi

**Affiliations:** 1 Department for musculoskeletal rehabilitation, Clinique romande de réadaptation suvacare, 90 avenue du grand champsec, Sion 1951, Switzerland; 2 Institut de Recherche en Réadaptation (IRR), Clinique romande de réadaptation suvacare, 90 avenue du grand champsec, Sion 1951, Switzerland

**Keywords:** CRPS1, Partial form, Hand-Budapest criteria

## Abstract

**Background:**

The partial form of the complex regional pain syndrome of the hand type 1 (CRPS 1), involving only 1 to 3 fingers, is a rare condition first described in 1972. The aim of the study is to define more precisely the diagnosis workup and the prognosis of this clinical entity.

**Methods:**

Retrospective study of CRPS1 partial form observed during five years in a rehabilitation ward. Application of The Budapest criteria, evaluation of radiological exams, therapeutic results and vocational outcomes. Comparison with cases from literature review.

**Results:**

132 patients were hospitalized with the diagnosis of CRPS type 1 of the hand. 16 partial forms were isolated: 11 men, 5 women with a mean age of 43 years. Among these patients, 14 (88%) met The Budapest criteria and the two remaining cases were diagnosed by using the three phase bone scintigraphy. Only moderate improvement was obtained in the majority of the patients. At the maximal time of follow-up (4 to 9 years), 50% of the patients hadn’t returned to work. From the literature review, 19 cases were eligible for clinical comparisons. The main differences between our series and the literature were: more men involved, later diagnosis and worst prognosis in term of return to work.

**Conclusions:**

This is the largest series of consecutive partial form of CRPS. The Budapest criteria are sufficient for the diagnosis in 88% of cases. As in complete form of CRPS1 of the hand, three phase bone scintigraphy should only be used in doubtful cases in the first six months of the illness. Partial form of CRPS1 of the hand is rare and its prevalence remains unknown. Long term prognosis (4 to 9 years) is poor in our series, 50% of patients didn’t returned to work.

## Background

Complex regional pain syndrome type 1 (CRPS1) most commonly involves the extremities and the hand in particular [1,2]. The clinical form that involves the whole hand is the most familiar. Diagnosis is generally straightforward. Partial forms involving one to three fingers have been described. Dammann [3] was the first to report three cases in 1972 which he called “fingers isolated Sudeck’s syndrome”. In 1977 and 1979, Lequesne et al. [4,5] described a form of partial algodystrophy termed “radial”, which follows a metameric topography on one or two rays of the hand or foot. Since then, a dozen articles have been devoted to the subject [6-16]. However, various diagnostic criteria were used in the literature and no clear diagnosis process for partial CRPS of the hand was given. The aims of our study are to present this clinical form in a patient population with CRPS1 of the hand, to assess whether the criteria developed by Harden et al. [17,18], the so called Budapest criteria, can be applied to this particular form and to define whether the various radiological examinations are still useful in order to propose an accurate diagnosis process for this rare entity. The results of this series are also compared to the data obtained from a literature review.

## Methods

### Population and diagnostic criteria

This is a retrospective single-centre study of patients admitted to a tertiary rehabilitation centre between January 2004 and December 2009. This study was approved by the regional medical ethics committee (*commission cantonale valaisanne d’éthique médicale*) with the reference number 043/07. All the patients signed an informed consent form for their participation and publication of images. The majority of patients were admitted to our hospital at the request of the leading Swiss accident insurer. Most patients were men employed in industry and the building trade with persistent deficiencies as a result of an industrial, road traffic or sporting accident. Patients with a partial form involving a maximum of 3 rays [15,16] were selected from those with complete CRPS1 of the hand. All our patients satisfied the French criteria for CRPS1 (algodystrophy) [1]. These criteria were in used in our hospital before Budapest’s criteria. The other inclusion criteria were: age between 18–65 years, no invalidity pension, no central nervous system lesion or significant lesion of the peripheral nervous system (CRPS type 2). The criteria developed in Budapest [17] for CRPS, validated in 2010 [18], were used and applied retrospectively to our series through the computer medical record which prospectively collects clinical data since 1999. The socioeconomic and other clinical data were also collected from this system. CRPS was treated according to current guidelines [19,20].

### Radiological data

The radiological data were gathered from available examination reports and images. For standard radiography, an anteroposterior view of both hands on the same film was used as the reference image [21]. The radiographic features of CRPS have been described in detail by Doury [1]: diffuse or peri /juxta-articular demineralisation, mottled demineralisation, predominantly subchondral bone resorption, endosteal resorption of the intracortical bone which may occur in combination according to the stage of CRPS. These signs and their localisation were noted for each file. For three-phase bone scintigraphy (TBPS), the scintigraphic classification of CRPS into 3 stages [22-29] was used: stage 1, early increased vascular and tissue perfusion and early and delayed bone hyperfixation; stage 2, absence of early hyperperfusion and delayed hyperfixation and stage 3, early tissue hypoperfusion, normalisation of bone fixation. The criteria described by Schürmann et al. [30] were used for magnetic resonance imaging (MRI): bone marrow oedema in a spot pattern of the carpal bones, subcutaneous oedema, subcutaneous and/or synovial contrast uptake during Gadolinium injection and joint effusion.

### Evaluation of the results of treatment and of course

During hospital stay, pain severity was assessed on admission and discharge using a visual analogical scale (VAS) from 0 to 100 mm (0 = no pain; 100 = the worst possible pain) [31,32]. A beneficial treatment effect on pain was considered to be present if the baseline value was reduced by at least 30% between admission and discharge [31]. Hand function was assessed using the Disabilities Arm Shoulder and Hand (DASH) questionnaire at the start and end of the hospital stay [33]. A reduction of at least 12.75 points on DASH between the start and end of the hospital stay defines the minimum detectable change of the DASH, that is to say the change observed which cannot be attributed to measurement error with a 95% confidence interval [33]. The perceived overall efficacy of treatment was also assessed on the basis of an external criterion [31]. The patient completed a VAS of overall treatment efficacy at discharge (0 mm = no effect; 100 mm = the greatest possible beneficial effect). The patient was considered to be improved if an efficacy of more than 30 mm on the VAS was detected, which corresponds to the smallest clinically significant change [31]. The results of these measures (VAS, DASH, overall treatment efficacy) are presented as the mean and percentage of patients considered being responders. The outcome at the maximal follow-up (4 to 9 years after hospitalization) was evaluated with the insurance records.

### Literature review

The articles in English, French and German published between 1970 and 2012 that dealt with partial CRPS 1 were searched with Pubmed and the reference lists of the articles identified. The following key words were used: algodystrophy, reflex sympathetic dystrophy, Sudeck’s atrophy, transient osteoporosis, CRPS, partial, segmental, hand. Exclusion criteria were as follows: review articles, articles in a language other than French, English and German, articles without a useful description of the cases and articles on CRPS type 2.

The following data were compared with the cases reported in the literature: sex, age, trigger factors, diagnostic delay, number of fingers affected, symptoms, clinical signs, standard radiography, TBPS and evolution.

## Results

### Population and clinical criteria

**Figure 1:**
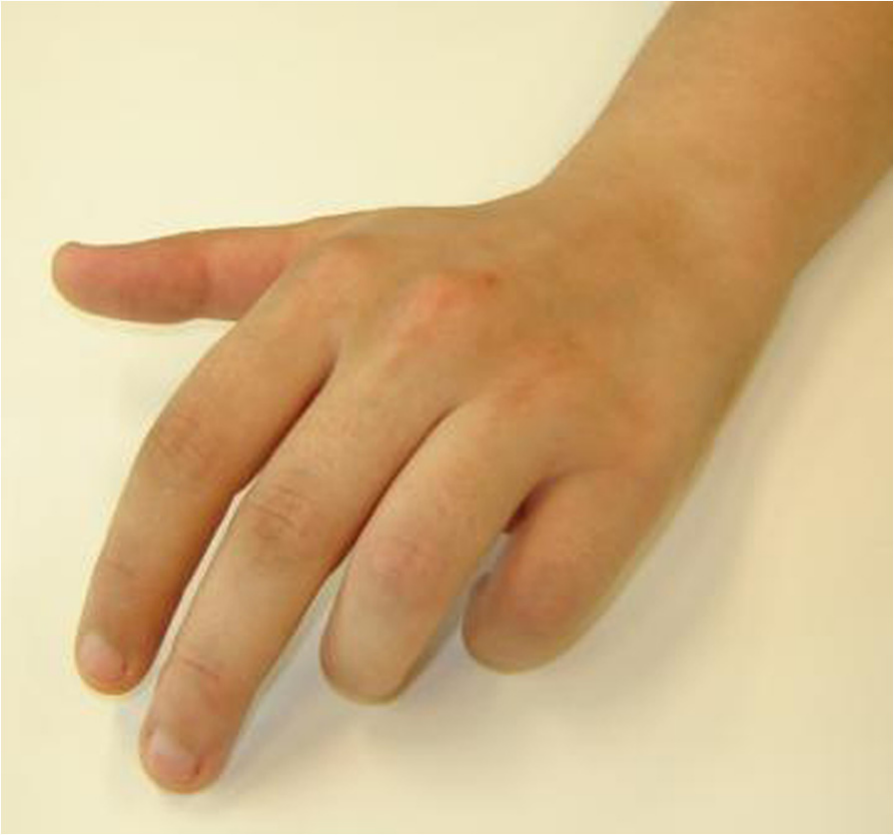
**Clinical aspect:23-year old female patient, contusion of the hand, development of pain and attitude of contracture that was partly reduced in the 4**^**th **^**and 5**^**th **^**fingers of the left hand.**

Tables 1 and 2 summarizes the data. In the study period, 132 patients were admitted with the diagnosis of CRPS of the hand. Of these 132 patients, 16 cases of partial forms of CRPS involving three rays at the maximum were selected. All fulfilled the French criteria [1]. The cases involved 11 men and 5 women with a mean age of 43 years, admitted for 30 days on average. Of these 16 cases, 14 fulfilled all the Budapest clinical diagnostic criteria [17,18]. With regard to the criteria (see Table 2), 4 symptoms were present in more than 50% of our patients: continuous pain, reduced mobility, hyperaesthesia/allodynia and oedema. Six clinical signs were also present in more than half: reduced mobility, change/asymmetry in colour, trophic disorders, change/asymmetry in sweating, hyperaesthesia/allodynia and oedema. Motor dysfunction was observed in 4 patients: exclusion of the thumb from function with thumb-in-palm (2 cases), thumb in permanent extension (1 case) and permanent reducible passive flexion in 4^th^ and 5^th^ fingers (1 case) (see Figure 1). During the hospitalization, two motor dysfunctions improved and the two others didn’t.

**Table 1:** Description of the two populations: demographic characteristics, triggering factors, initial injuries and outcome

	**Our series (n = 16)**			**Literature (n = 19)**		
**Sex-ratio**	**11 M/5 W**			**8 M/ 11 W**		
**Average age**	**43 years (25 à 59 years)**			**48,1 years (18 à 71 years)**		
**Triggering factors**	**Traumatic**	**16**	**100.0%**	**Traumatic**	**14**	**74%**
	**Non traumatic**	**0**	**0%**	**Non traumatic**	**5**	**26%**
**Initial injury**	**Contusion/sprain**	**2**	**13%**	**Contusion/sprain**	**6**	**32%**
	**Fracture/dislocation**	**5**	**31%**	**Fracture/dislocation**	**4**	**21%**
	**Tendinous injury**	**4**	**25%**	**Tendinous injury**	**0**	**0%**
	**Superficial wounds**	**0**	**0%**	**Superficial Wounds**	**4**	**21%**
	Complex injury*	**2**	**13%**	Complex injury*	**0**	**0%**
	**Others**	**3**	**19%**	**Others**	**5**	**27%**
**Diagnosis extension (days)**	**Average**	**207**		**Average**	**81 (n = 15)**	
	**Median**	**149**		**Median**	**Unavailable**	
**Mean number of sick leave days compensate**	**Before admission (n = 16)**	**202 (62 à 501)**		**Unavailable**		
	**After discharge (n = 14)**	**463 (13 à 1147)**				
**Outcome at the maximal endpoint**	**Change of work**	**3**	**19%**	**Complete recovery (12 to 16 months average = 6 months)**	**8**	**42%**
**Our series n = 15**	**Return to same work**	**4**	**25%**	**Incomplete improvement**	**6**	**32%**
**Literature n = 16**				**(6 to 12 months average = 8 months)**		
	**No activity (unemployment, social security …)**	**8**	**50%**	**Permanent disability**	**2**	**10%**
	**Lost to follow up**	**1**	**6%**	**unavailable**	**3**	**16%**
	**Invalidity pension (Severe brain injury)**	**1**				

* Complex injury: injury of bone + soft tissues + nerve and/or vessels.

**Table 2:** Clinical and radiological characteristics of the two populations

	**Our series (n = 16)**		**Literature (n = 19)**	
**Clinical data**	**N**	**%**	**N**	**%**
**(Budapest criteria)**				
**Symptoms**				
**Continuing pain disproportionate**	**16**	**100**	**14**	**74**
**Decreased range of motion**	**16**	**100**	**3**	**16**
**Hyperesthesia and/or allodynia**	**11**	**69**	**6**	**32**
**Edema**	**9**	**56**	**4**	**21**
**Changes/asymmetry skin colour**	**7**	**44**	**2**	**11**
**Changes/asymmetry sweating**	**6**	**38**	**0**	**0**
**Temperature asymmetry**	**4**	**25**	**1**	**5**
**Trophic changes (hair, nail, skin)**	**3**	**19**	**0**	**0**
**Motor dysfunction**	**3**	**19**	**0**	**0**
**Clinical signs**				
**Decreased range of motion**	**16**	**100**	**17**	**90**
**Changes/asymmetry skin colour**	**12**	**75**	**9**	**47**
**Trophic changes**	**10**	**63**	**1**	**5**
**Hyperesthesia and/or Allodynia**	**10**	**63**	**10**	**53**
**Edema**	**9**	**56**	**10**	**53**
**Changes/asymmetry sweating**	**9**	**56**	**4**	**21**
**Temperature asymmetry**	**6**	**38**	**5**	**26**
**Motor dysfunction**	**4**	**25**	**0**	**0**
**Number of fingers injured**				
**1**	**7**	**44**	**10**	**52**
**2**	**2**	**12**	**7**	**37**
**3**	**6**	**38**	**2**	**11**
**2 bilateral**	**1**	**6**	**0**	**0**
**Radiographics data**				
**Standard roentgenogram**	**14**	**88**	**11**	**58**
**Normal**	**7**	**50**	**2**	**18**
**Diffuse demineralization of injured digits**	**6**	**43**	**8**	**72**
**Global Peri articular demineralization of all digits**	**1**	**7**	**1**	**10**
**Three-phase bone scaning**	**16**	**100**	**11**	**58**
**Available**	**15**	**94**	**11**	**58**
**No CRPS**	**3**	**20**	**0**	**0**
**CRPS Stage 1 et 1/2**	**7**	**47**	**11**	**100**
**CRPS Stage 2 et 2/3**	**4**	**27**	**0**	**0**
**CRPS Stage 3**	**1**	**6**	**0**	**0**

### Radiological examinations

The data are summarised in Table 2. Standard radiography of both hands in the anteroposterior view was performed on the same film in 14 patients (88%). Radiography showed localised demineralisation of the affected ray(s) in 6 patients (43%). The demineralisation appeared mottled in 2 cases only. TBPS was performed on the 16 patients, but was available for 15 only. It was performed 220 days (median 155 days) on average after the initial accident. TBPS was performed during hospital stay in 87% of cases by the same specialist. In 12 cases (80%), TBPS supported the presence of CRPS. When it was performed before 6 months (53% of cases), six cases of stage 1 and two of stage 2 were found. When TBPS was performed (7 cases) more than 6 months after the trauma, the images did not support CRPS (3 cases) or were consistent with stage 2/3 or 3 (2 cases). In only 2 cases there was a suggestion of stage 1 or 2. In the two patients who did not meet the Budapest criteria for CRPS, TBPS was clearly in favour of CRPS stage 1. The MRI of the hand, performed 4 times, provided no evidence to support CRPS in 3 cases out of 4. In the last case, carpal oedema was associated with synovial thickening causing contrast uptake, 1 month after the accident, compatible with incipient CRPS.

### Evaluation of treatment’s results and evolution

The pain on a VAS at admission and discharge was available for 14 of the 16 patients (88%). Mean pain on the VAS on admission was 55 mm and on discharge was 43 mm. Seven patients (47%) showed at least 30% improvement compared to the admission VAS (from 59 mm to 29 mm on average). The DASH questionnaire on admission and discharge was available for 12 patients with a mean score of 55/100 on admission and 51/100 on discharge giving a mean reduction of −4 points. Only 3 patients (25%) had an admission/discharge difference greater than 12.75 points. The VAS “patient beneficial treatment effect” was available for 14 patients (mean of 59 mm). In 11 cases (79%) it was greater than 30 mm (from 37 to 100 mm). With regard to professional activity, 4 to 9 years after the hospitalization, 50% of patients hadn’t returned to work, but we didn’t know the exact reason. 44% returned to the same job or an adapted job. One patient had an invalidity pension because of another injury (severe cranial trauma). The mean compensation duration was 202 days before and 463 days after hospitalization.

### Literature review

Fourteen articles were identified [3-16]. Half of these were excluded [4,5,7-9,14,15]. Two articles presented case series but without details of the clinical findings and thus could not be used in this review [8,15]; four articles were about CRPS type 2 [4,5,7,14] and the case in the last article was not convincing [9]. Seven articles were finally included [3,6,10-13,16] involving 19 cases. The comparative sample consists of these articles. The diagnostic criteria used, when given, varied (Doury, Amadio, Veldman) and no study applied the Budapest criteria. The diagnosis of CRPS was assessed on the basis of the clinical and radiological descriptions in the articles. The literature data are summarised in Tables 1 and 2. Compared to our series, there were more women and 75% of cases were post-traumatic in origin. Mean diagnostic delay was much shorter (2.7 months versus 7.5 months). The main symptoms were pain, hyperaesthesia/allodynia and oedema. The clinical signs present in more than 50% of cases were reduced joint mobility, hyperaesthesia/allodynia and oedema. Standard radiography was performed in 58% of the cases and demonstrated demineralisation of the affected ray(s) in 72% of cases. TBPS was performed in 52% of cases. It was always considered to support the diagnosis of CRPS, but only data from the delayed phase were described. No data on MRI were available.

With regard to evolution, 8 patients (42%) were cured and 6 (31%) improved with an extension of 6 to 12 months. Two patients were in permanent disability at 1.5 and 9 years.

## Discussion

This series is the largest well-documented consecutive series of partial CRPS 1 of the hand. It is also the first to use the recently validated diagnostic criteria, the so called Budapest criteria [17,18], in this context. Among our 16 patients, 14 (88%) fulfilled these criteria and in the two remaining cases, where just one symptom was missing, a TBPS enabled the diagnosis to be confirmed. Validated criteria accepted by the majority of the medical community make the comparison of studies and grouping of data from small series possible, which facilitates the advancement of knowledge in rare diseases. It should be kept in mind that there is still no consensus in the literature on the number of rays that define the partial form of CRPS. Doury and Lequesne et al. [1,4,5] proposed that one or two fingers at most must be affected. However, Soucacos et al. [15] and Bianchi et al. [16] proposed three fingers. Based on our experience, we propose to apply the diagnosis of partial CRPS when one to three rays are affected and when the disease does not spread to the whole hand later as part of overall CRPS progression. Some authors discussed a neurological origin of partial forms of CRPS [6,12,13,16] but they gave no evidence to support this hypothesis.

In these partial forms of CRPS of the hand, special importance should be given to differential diagnosis which meets the Budapest criterion n° 4 [17,18] i.e. that no other diagnosis may better explains the signs and symptoms. For the hand, many other diagnoses should be excluded [1,4,5,10]. Radiological examinations may therefore remain useful especially for doubtful or borderline cases. It would be appropriate then to specify their importance when partial involvement is suspected.

Based on the available data, standard radiography and MRI have limited value. They should not be performed for confirming partial CRPS of the hand, but only for differential diagnosis [21,29,30].

**Figure 2:**
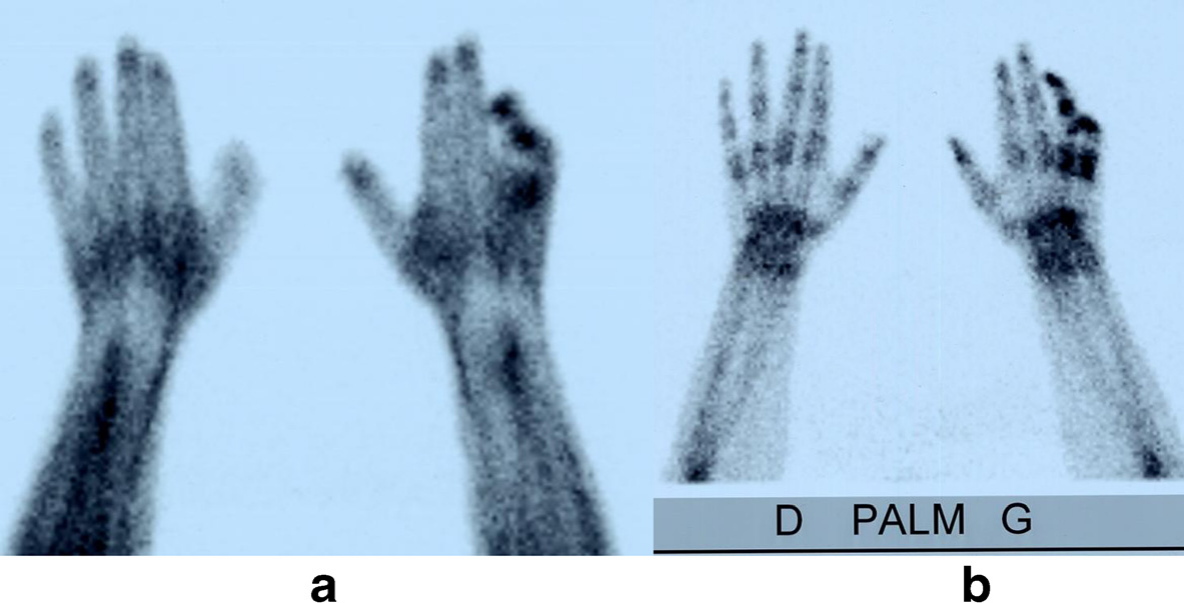
**Three-phase bone scintigraphy: early phase (a) and delayed phase (b) Same patient as Figure **1**.** Staged early and delayed hyperfixation on 4^th^ and 5^th^ fingers suggesting CRPS stage 1.

**Figure 3:**
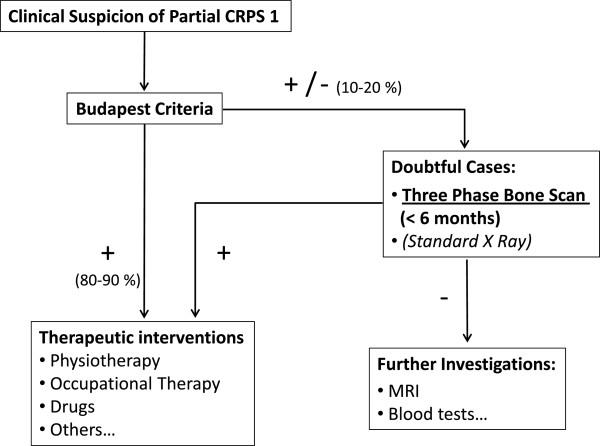
Partial CRPS type 1 of the hand: proposed diagnostic flow chart.

TBPS has been used since the early eighties in CRPS for the upper limb [22-27], but still not for the partial forms of CRPS. A review article published in 1995 [28] concluded that TBPS was the most useful for positive diagnosis of CRPS when performed within the first 6 months of the disease. In another hand, in Dutch guidelines published in 2006 [34], TBPS was considered to have no additional diagnostic value. Since the publication of these guidelines, two meta-analysis were recently published [29,35] with contradictory results. Moreover, 3 prospective studies have been also published [26,27,30] confirming the usefulness of TBPS as additional tool in doubtful cases [30] and when the duration of the pathology is shorter than 3 [26] or 5 months [27]. Two other retrospective studies [36,37] using the Budapest criteria were also recently published. Moon et al. [36], found a low utility of TBPS for diagnosis of CRPS and AlSharif et al. [37] found a positive scintigraphy in patients with vasomotor symptoms, motor and/or trophic changes, with a duration of less than 3 months. In our study, TBPS was also particularly helpful when it was performed in the first 6 months. Figure 2 shows the TBPS images in a partial form. Taking all these results together, the role of TBPS in the diagnosis of CRPS is still uncertain. It is certainly not a screening tool for diagnosis of CRPS in which clinical findings remain the gold standard. Nevertheless, we believe that TBPS should be only recommended in the first months of the disease progression for unclear situations which do not fully meet the Budapest criteria [29,30]. Based on the present knowledge, we have proposed a diagnosis flow chart (Figure 3) on the application of the Budapest criteria and of radiology in cases of partial CRPS.

The disease course, reported briefly in the literature, is described as favourable in the majority of cases (see Table 1), but return to work is addressed in only 2 patients (10%). In relation to our patients, progress during their hospital stay was modest. Nearly half of our patients (47%) reported improvement of at least 30% in pain, which is considered the smallest clinically significant change that could be detected [31]. The patients’ perception of global improvement was more than 50 mm and can be defined as clinically significant change (i.e. more than 30 mm) in 80% of patients. This more global parameter probably reflects patient satisfaction with the whole interdisciplinary rehabilitation process. Regarding return to work, in our series, 50% of patients didn’t return to work with a follow up of 4 to 9 years. We cannot explain this long lasting sick leave but none of our patients had an invalidity pension related to CRPS. The literature is not very precise, especially concerning return to work, and to date our study is the first to use detailed insurance data. Hence, it could happen that old studies may have been more optimistic than those performed more recently [1,38]. But it is also possible that a too late diagnosis, as in our cases, may have had a negative impact on healing, especially on chronic pain. Finally, it could not be excluded that cases with favourable outcomes were not sent to our centre.

Compared to the results of literature, our patients are all post traumatic with a higher prevalence of males. This is due to the selection of our patients, i.e. most of them were blue collars (industry and building workers) insured to the leading Swiss insurance company which owns our clinic (selection bias). Then the diagnosis of CRPS was made more belatedly and the prognosis seems worse in our series. This probably due to the fact that this syndrome is rare, ignored by the majority of medical practitioners and discovered in specialised wards [3-16]. In our medical environment, traumatic patients are only sent to tertiary centres in case of unfavourable outcome after months what probably explains this late diagnosis (202 days on average).

Different diagnostic sets were used in the literature. This may also affect comparisons with our series [39,40]. Nevertheless, with the exception of motor dysfunction, only recently introduced in CRPS criteria, all the other symptoms and signs were somehow already mentioned in former classifications and used in hand rehabilitation facilities for many decades [1,2]. Moreover, we have only kept studies with precise clinical description. For these reasons, we assume that all these cases are partial form of CRPS. Computer medical records are very helpful to trace these criteria. In our hospital, for instance, clinical data are prospectively recorded and were used as database for all CRPS cases since 2002 for the lower limb [41] and 2004 for the upper limb with only few missing data.

The first limitation of our study is its retrospective nature. With this design, we can’t exclude a placebo effect to explain a part of therapeutics results [42]. Because symptoms can also fluctuate over time [40], it is also possible that retrospective design with only one assessment may present a low precision of the measurement. The third limitation is related to the selection bias i.e. mostly blue-collar male patients with unfavourable outcome are hospitalized in our clinic (see above). For these reasons, our results cannot be generalised to all cases of partial CRPS. Finally, the literature review remains limited by the diversity of diagnosis criteria, but the articles were carefully selected with precise clinical description and were assumed to be partial forms of CRPS.

## Conclusions

In conclusion, partial CRPS type 1 of the hand is a rare clinical form. For clinical practice we recommend the use of the Budapest criteria validated in 2010 [18], with a maximum of 3 rays involved without subsequent spread. Only, in case of doubt or borderline form (10 to 20% of cases), TBPS should be performed in the first six months of the disease course after trauma, other radiological examinations being devoted to differential diagnosis above all. In our series, the prognosis is poor, 50% of patients didn’t return to work 4 to 9 years after hospitalization.

## Abbreviations

CRPS: Complex regional pain syndrome; TBPS: Three bone phase scintigraphy or scan; MRI: Magnetic resonance imaging; VAS: Visual analogical scale; DASH: Disabilities arm shoulder and hand; IASP: International association for study of pain.

## Competing interest

The authors declare that they have no competing interest.

## Authors’ contribution

MK extracts the patient’s data, analyzes it, wrote the article, and made the tables and figures. OD participated in the redaction of some sections of the revise manuscript in English. FL participated in the redaction, correction and review of this article. All authors read and approved the final manuscript.

## Pre-publication history

The pre-publication history for this paper can be accessed here:

http://www.biomedcentral.com/1471-2377/13/28/prepub
